# Detection and characterization of chicken anemia virus from commercial broiler breeder chickens

**DOI:** 10.1186/1743-422X-5-128

**Published:** 2008-10-27

**Authors:** Zerihun Hailemariam, Abdul Rahman Omar, Mohd Hair-Bejo, Tan Ching Giap

**Affiliations:** 1 Faculty of Veterinary Medicine, Haramaya University, P.O. Box 271, Haramaya, Ethiopia; 2 Institute of BioScience, Universiti Putra Malaysia, 43400 UPM Serdang, Selangor Darul Ehsan, Malaysia; 3 Faculty of Veterinary Medicine, Universiti Putra Malaysia, 43400 UPM Serdang, Selangor, Darul Ehsan, Malaysia

## Abstract

**Background:**

Chicken anemia virus (CAV) is the causative agent of chicken infectious anemia (CIA). Study on the type of CAV isolates present and their genetic diversity, transmission to their progeny and level of protection afforded in the breeder farms is lacking in Malaysia. Hence, the present study was aimed to detect CAV from commercial broiler breeder farms and characterize CAV positive samples based on sequence and phylogenetic analysis of partial VP1 gene.

**Results:**

A total of 12 CAV isolates from different commercial broiler breeder farms were isolated and characterized. Detection of CAV positive embryos by the PCR assay in the range of 40 to 100% for different farms indicated high level of occurrence of vertical transmission of viral DNA to the progeny. CAV antigen was detected in the thymus and in the bone marrow but not in spleen, liver, duodenum, ovary and oviduct by indirect immunoperoxidase staining. The 12 CAV isolates were characterized based on partial sequences of VP1 gene. Six isolates (MF1A, MF3C, M3B5, NF4A, P12B and P24A) were found to have maximum homology with previously characterized Malaysian isolate SMSC-1, four isolates (M1B1, NF3A, PYT4 and PPW4) with isolate BL-5 and the remaining two (NF1D and NF2C) have maximum homology both with isolates 3-1 and BL-5. Meanwhile, seven of the isolates with amino acid profile of 75-I, 97-L, 139-Q and 144-Q were clustered together in cluster I together with other isolates from different geographical places. The remaining five isolates with amino acid profile of 75-V, 97-M, 139-K and 144-E were grouped under cluster II. All the CAV isolates demonstrated omega values (K_a_/K_s_) of less than one (the values ranging from 0.07 to 0.5) suggesting the occurrence of purifying (negative) selection in all the studied isolates.

**Conclusion:**

The present study showed that CAV is widespread in the studied commercial broiler breeder farms. The result also indicated the occurrence of genetic variability in local CAV isolates that can be divided at least into two groups based on characteristic amino acid substitutions at positions 75, 97, 139 and 144 of the VP1 protein.

## Background

Chicken anemia virus (CAV) is a small DNA virus with a circular, covalently linked, single negative-strand genome. It is the causative agent of chicken infectious anemia (CIA) and classified in the family Circoviridae, genus Gyrovirus [1].

CAV is an economically important pathogen with a world-wide distribution. CAV infections are manifested by either clinical or subclinical signs [2]. The clinical disease is mainly noticed in young chicks of 10–14 days of age, which usually acquire the infection vertically. Chickens older than 2–3 weeks of age are also susceptible to infection, but will only develop a subclinical disease evidenced by poor vaccine response, increased severity of other infections [2,3], and decreased cell mediated immune responses [4,5]. Outbreaks of the disease are characterized by anemia, thymus atrophy, bone marrow aplasia and immunosuppression [3,6].

In general, no significant antigenic or pathogenic difference was reported among the CAV isolates in the past. Thus, until lately, CAV was known as a much conserved virus of one serotype [7] with several genetic groups [8]. However, an antigenically different isolate (CAV-7) has been reported from USA [9,10], which could be a prototype virus of serotype 2. In Malaysia, previous studies undertaken indicated high prevalence of the virus in commercial broiler and layer farms [11]. Subsequently, CAV isolates were isolated from broilers farms and some of these isolates have been characterized based on pathogenicity and molecular analysis [12,13]. However, there is no study conducted in the broiler breeder farms regarding the extent of occurrence of the virus, type of isolates present and their genetic diversity. In the present study we report detection of CAV and characterization of isolates based on sequence and phylogenetic analysis of partial VP1 gene from commercial broiler breeder chickens in Malaysia. Level of transmission to the progeny and level of protection afforded in the commercial broiler breeder chickens were also analyzed and discussed.

## Results

### Distribution of CAV DNA in various organs in commercial broiler breeder hens

A total of 420 organ samples collected from 60 commercial broiler breeder hens were tested by nested PCR assay for the presence of CAV DNA. The data are summarized in Additional file 1. The highest percentage of positive samples was detected in spleen where 45 samples out of 60 (75%) were positive for CAV DNA. Duodenum was found to be an organ with the least distribution of CAV DNA in which 28 organs out of 60 (46.7%) were positive for CAV DNA. Even though, there is difference in the percentages of CAV DNA between spleen, bone marrow, thymus and ovary, the differences were not statistically significant (P < 0.05). However, the distributions of viral DNA in liver, duodenum and oviduct were significantly less (P < 0.05) from the rest of the organs.

### CAV DNA in embryos and egg shell membranes (ESM)

**Figure 1:**
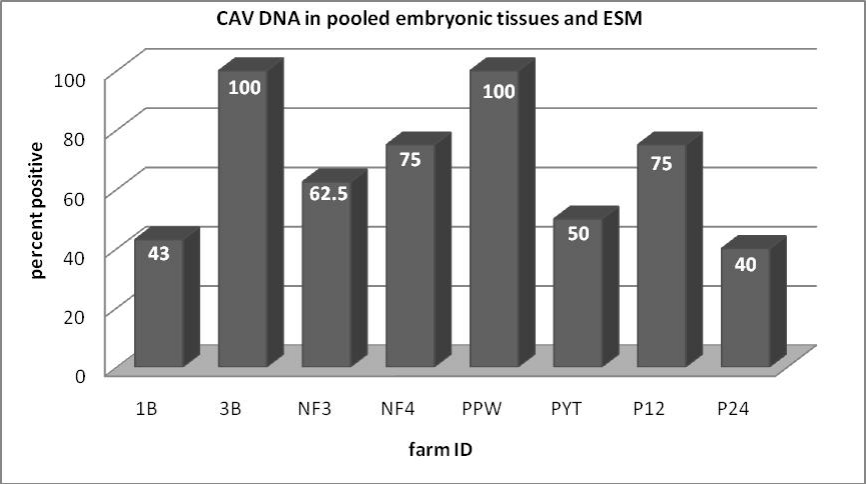
**Detection of CAV DNA in pooled embryonic tissues and ESM from eggs collected from commercial broiler breeder farms**. As it is shown on the graph, CAV DNA was detected in 40 to 100% of pooled embryonic tissue and ESM samples tested for different commercial broiler breeder farms.

The nested PCR assay result indicated the presence of positive embryos ranging from 40–100% in different farms from three states of Malaysia (Fig. 1).

### Nucleotide sequence analysis

Nucleotide sequence analysis of a 498 bp region of CAV genome from position 892 to 1389; numbering according to Meehan *et al*. [14], encompassing the hypervariable region of VP1 protein revealed total nucleotide variation among the isolates ranging from 0.3 to 6.1% whilst the overall maximum nucleotide variation is 6.5%. The nucleotide sequence alignment with the published isolates considered for comparison revealed 9 to 30 nucleotide substitutions in the isolates from commercial broiler chickens (Table 2). Based on comparisons of percentage homologies, six isolates (MF1A, MF3C, M3B5, NF4A, P12B and P24A) were found to have maximum homology (97.1 to 99.1%) with SMSC-1 isolate, four isolates (M1B1, NF3A, PYT4 and PPW4) were found to have maximum homology (98.1 to 98.9%) with BL-5 isolate and the remaining two (NF1D and NF2C) have similar maximum homology (98.1%) both with isolates 3-1 and BL-5 (Table 1).

**Table 1:** Nucleotide percentage homologies of studied isolates from the commercial broiler breeder hens in relation to previously characterized Malaysian CAV field isolates.

	Previously identified Malaysian CAV isolates		
	
	SMSC-1	BL-5	3-1
MF1A	**98.9**	94.9	94.5
MF3C	**97.9**	94.5	94.3
M3B5	**97.1**	95.1	94.9
NF4A	**98.3**	95.1	94.7
P12B	**99.1**	95.1	95.1
P24A	**98.7**	95.1	94.7
M1B1	94.9	**98.9**	98.1
NF3A	94.1	**98.5**	97.7
PYT4	94.1	**98.5**	97.7
PPW4	94.7	**98.1**	97.1
NF1D	94.7	**98.1**	**98.1**
NF2C	94.3	**98.1**	**98.1**

Accordingly, isolates MF1A, MF3C, M3B5, NF4A, P12B and P24A were found to have maximum homology with SMSC-1 isolate, where as four isolates (M1B1, NF3A, PYT4 and PPW4) were found to have maximum homology with BL-5 isolate. However, isolates NF1D and NF2C have similar maximum homology (98.1%) both with isolates 3-1 and BL-5.

**Table 2:** Number of nucleotide and amino acid substitutions and Ka/Ks ratio of Malaysian CAV isolates

CAV isolates	*No. of Nucleotide Substitutions	*No. of amino acid Substitutions	non-synonymous substitution rate (*K*_*A*_)	Synonymous substitution rate (*K*_*S*_)	Omega value (*K*_*A*_/*K*_*S*_)	References
MF1A	28	6	0.017	0.199	0.08	This study
MF3C	30	6	0.017	0.225	0.07	This study
M3B5	27	6	0.017	0.191	0.09	This study
NF4A	27	7	0.020	0.179	0.11	This study
P12B	27	6	0.017	0.187	0.09	This study
P24A	18	6	0.017	0.191	0.09	This study
PPW4	12	7	0.020	0.039	0.50	This study
NF1D	13	6	0.017	0.053	0.33	This study
NF2C	13	6	0.017	0.055	0.31	This study
NF3A	11	4	0.012	0.047	0.25	This study
PYT4	11	5	0.015	0.042	0.35	This study
M1B1	9	4	0.012	0.036	0.32	This study
SMSC-1	26	5	0.017	0.187	0.09	[13]
3-1	8	4	0.011	0.060	0.19	[13]
BL-5	4	3	0.009	0.006	1.50	[12]

* compare to the consensus nucleotide and amino acid sequences. Pair wise comparison with the consensus nucleotide and amino acid sequence indicated Ka/Ks ratio less than one for all the studied isolates which indicates the occurrence of negative selection.

Compared to isolates from other geographical places around the world, six out of 12 isolates (MF1A, MF3C, M3B5, NF4A, P12B, and P24A) were found to have maximum homology with Australian isolate 704. The remaining isolates showed maximum homology with isolates from China (NF3A and PYT4), isolate A2 from Japan (NF1D and NF2C) and CAV-B isolate from India (PPW4). Only two of the isolates (M1B1 and NF3A) were found to have maximum homology with isolates 26P4 and Del-Ros from USA.

### Amino acid sequence analysis

The amino acid sequence alignment with the published isolates considered for comparison also showed 4 to 7 amino acid substitutions in the isolates from commercial broiler breeder chickens (Table 2). The calculation of synonymous and non-synonymous substitution rate demonstrated omega values (K_a_/K_s_) of less than one suggesting the occurrence of purifying (negative) selection in all the 12 isolates (Table 2). All the isolates have the omega value ranging from 0.07 to 0.35 except for PPW4 with omega value of 0.50. Eight variable amino acid positions were detected in more than one isolate at amino acid positions 22, 75, 83, 97, 125, 139, 141, 144. Maximum variation among the CAV isolates was observed at amino acid position 144. Proline (P) at position 22 for isolate NF4A and glycine (G) at position 48 for isolate PYT4 were unique amino acid substitutions found only in the studied isolates (Table 3).

**Table 3:** Amino acid substitutions in VP1 sequence of CAV isolates

Isolate	Amino acid positions									
	
	22	48	75	83	97	125	139	141	144	157
Consensus	H	A	V	I	M	I	K	Q	Q	V

M1B1	.	.	.	.	.	.	.	.	E	M
NF1D	.	.	.	L	.	L	.	E	E	.
NF2C	.	.	.	L	.	L	.	E	E	.
NF3A	.	.	.	.	.	L	.	.	E	.
PYT4	.	G*	.	.	.	L	.	.	E	.
NF4A	P*	.	I	.	L	.	Q	.	.	.
PPW4	N	.	I	.	L	.	Q	.	.	.
P24A	.	.	I	.	L	.	Q	.	.	.
P12B	.	.	I	.	L	.	Q	.	.	.
M3B5	.	.	I	.	L	.	Q	.	.	.
MF3C	.	.	I	.	L	.	Q	.	.	.
MF1A	.	.	I	.	L	.	Q	.	.	.
NIE/19.04/118/Nigeria	.	.	I	.	L	.	Q	.	.	.
BD-3/Bangladesh	.	.	I	.	L	.	Q	.	.	.
ISOLATE704/Australia	.	.	I	.	L	.	Q	.	.	.
130/Slovenia	.	.	I		L	.	Q	.	.	.
CAV-B/India	.	.	I	.	L	.	Q	.	.	.
SMSC-1/Malaysia	.	.	I	.	L	.	Q	.	.	.
BL5/Malaysia	.	.	.	.	.	.	.	.	E	.
BL5/P90/Malaysia	.	.	.	.	.	.	.	.	K	.
SMSC-1/P60/Malaysia	.	.	.	L	.	.	.	E	E	M
3-1/Malaysia	.	.	.	.	.	.	.	.	E	.
3-1/P60/Malaysia	.	.	.	.	.	.	.	E	E	M
CUX-1(M)/Germany	.	.	.	.	.	.	.	.	D	.
CUX-1(N)Germany	.	.	.	.	.	.	.	.	D	.
CIA-1/USA	N	.	I	.	L	.	Q	.	.	.
ConnB/USA	.	.	.	.	.	.	.	.	N	.
Del-Ros/USA	.	.	.	.	.	.	.	.	E	.
26P4/USA	.	.	.	.	.	.	.	.	E	M
CAV-A/India	.	.	.	.	.	L	.	.	D	.
A2/Japan	.	.	.	.	.	.	.	E	E	M
AF448446/China	.	.	.	.	.	.	.	.	E	.

Amino acids marked with an asterisk (*) are unique substitutions for isolates from commercial broiler breeder chickens. The isolates can be grouped into two distinct groups based on their amino acid profile at positions 75, 97, 139 and 144. Five isolates (M1B1, NF1D, NF2C, NF3A and PYT4) had amino acid profile of 75-V, 97-M, 139-K and 144-E.

### Phylogenetic analysis

**Figure 2:**
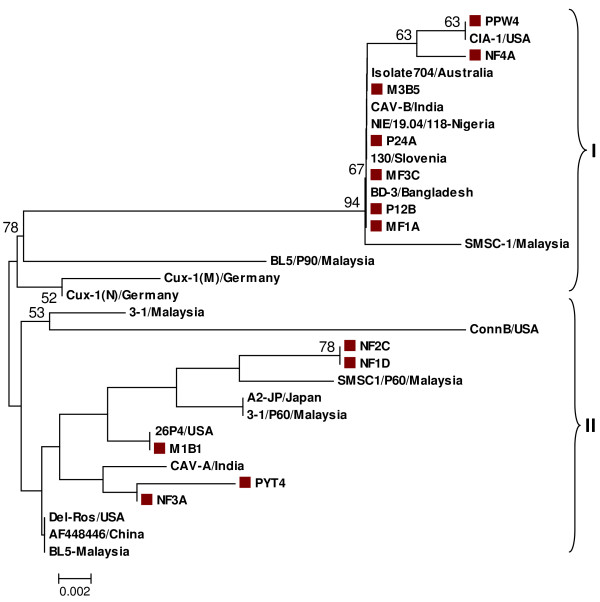
**Phylogenetic relationship among 32 different CAV isolates based on partial VP1 amino acid sequences. **Note: The boxes (■) indicate isolates identified in this study. The isolates were found both cluster I and II. Seven of the isolates with amino acid profiles of 75-I, 97-L, 139-Q and 144-Q clustered together in cluster I. The remaining five studied isolates with amino acid profiles of 75-V, 97-M, 139-K and 144-E were grouped under cluster II.

Phylogenetic analysis based on 165 deduced amino acid sequences of VP1 protein of the 12 isolates in comparison to 20 previously identified isolates revealed the formation of two clusters. All the 12 isolates were found both in cluster I and II (Fig. 2). Seven of the isolates with amino acid profile of 75-I, 97-L, 139-Q and 144-Q were clustered together in cluster I while the remaining five isolates with amino acid profile of 75-V, 97-M, 139-K and 144-E were grouped under cluster II.

### ELISA

**Figure 3:**
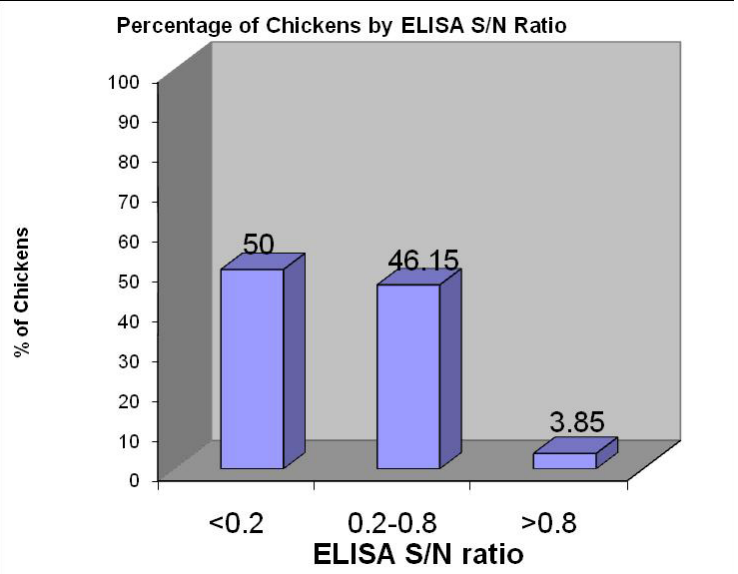
**ELISA results of serum collected from commercial broiler breeder chickens**. Fifty percent of the chickens have ELISA S/N < 0.2 which indicates the presence of high protective titers and able to afford high level protection to the progeny, meanwhile 46.15% of the chickens have ELISA S/N in the range of 0.2 to 0.8 affording low levels of protection to the progeny. Only 3.85% of the chickens have ELISA S/N > 0.8 indicating negative result for antibody against CAV.

The ELISA result shows that CAV infection is widespread in these unvaccinated commercial broiler chicken farms in Malaysia. Out of 52 chickens from which serum sample was collected, 50 (96.15%) were positive for antibodies against CAV (Fig. 3).

Out of the total number of hens sampled 26 hens (50%) have anti-CAV antibody titers above 8600 (high protective titers), 24 hens (46.15%) have the titer below 8600 but above 1000 (moderate protective titers) and 2 hens (3.85%) have anti-CAV antibody titers below 1000 (negative for antibodies against CAV). The ELISA-based analysis indicated that all farms had neutralizing antibodies. Based on the correlation of ELISA titer to virus neutralizing antibody titer as recommended by the manufacturer of the ELISA kit, 50% of hens found out to have high neutralizing antibody titers that able to confer high level protection to the progeny whilst 46.15% of hens have low neutralizing antibody titers affording low levels of protection to the progeny (Fig. 3).

### Indirect immunoperoxidase staining

**Figure 4:**
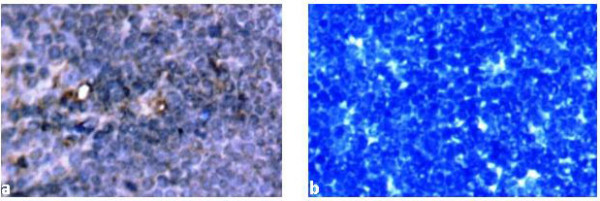
**IPS performed on formalin fixed paraffin-embedded thymic tissues**. Thymic tissue slide from commercial broiler breeder hen: a) infected lymphoblasts in the cortex demonstrated by brown staining (400×) b) IPS using CAV negative serum as primary antibody and devoid of any specific brown staining (400×).

**Figure 5:**
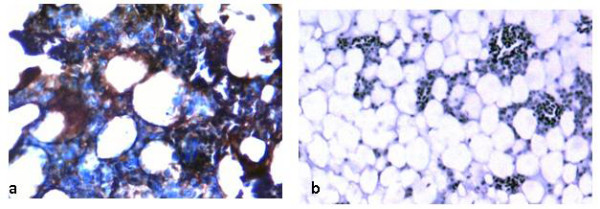
**IPS performed on formalin fixed paraffin-embedded tissues from bone marrow**. Bone marrow tissue slide from commercial broiler breeder hen: a) infected hemocytoblasts in the sinuses of the bone marrow demonstrated by brown staining (400×) b) IPS using CAV negative serum as primary antibody and devoid of any specific brown staining (200×).

All the experimentally infected chickens from hyperimmune serum production produced the highest readable antibody titer (>8661) which remained constant starting from two weeks after the second inoculation. Sera from the inoculated and control chickens were used as primary antibodies for the indirect immunoperoxidase staining. Specific positive staining was demonstrated in thymus and bone marrow tissue sections from commercial broiler breeder chickens that were detected positive for CAV DNA. Positive staining was observed in lymphoblasts in the cortex of the thymus (Fig. 4) and in hemocytoblasts in the sinuses of the bone marrow (Fig. 5). Specific staining was not demonstrated in tissue sections from spleen, liver, duodenum, ovary and oviduct.

## Discussion

In the present study, out of 420 organ samples tested, 75% of spleen, 68.3% of bone marrow, 70% of thymus, 53.3% of liver, 46.7% of duodenum, 66.7% of ovary and 48.3% of oviduct tested were positive for CAV DNA. CAV replicates mostly in lymphoid tissues of susceptible chickens [15-17]. Cardona et al. [18] found out a few CAV positive cells in spleen by *in situ *PCR and managed to detect CAV in ovaries by *in situ *PCR even in the absence of splenic virus. They also indicated that ovaries and to a lesser degree infundibulum of the oviduct are sites for persistence of CAV in hens. The finding of significantly higher positive tissues in the spleen, bone marrow, thymus and ovary as compared to duodenum, liver and oviduct in the present study is similar to the aforementioned findings. Therefore, we can suggest that spleen, thymus, and bone marrow could serve as an excellent choice of organs for the diagnosis of CAV infection while ovary representing more favourable tissue for the persistence of CAV in the reproductive organs in the broiler breeder hens.

Results from ELISA reading showed that 96.15% of blood samples collected from the same farms were positive for antibody against CAV indicating the widespread occurrence of CAV infection in these unvaccinated commercial broiler breeder chicken farms. Testing pooled embryonic tissue samples (thymus, bursa of Fabricius and spleen) together with ESM showed positive embryos for CAV DNA in the range of 40% to 100% for different commercial broiler breeder chickens despite the presence of neutralizing antibodies in majority of the hens (96.15%) tested for CAV antibodies suggesting high level of occurrence of vertical transmission of viral DNA to the progeny. The detection of CAV DNA in the ovary and oviduct of commercial broiler breeder hens with virus neutralizing antibodies and in their embryos supports the previous evidence that CAV may remain in the gonads of antibody positive chickens and can be vertically transmitted to their progeny [18-21]. The finding by Schat et al. [2] indicated low level of viral transcripts can be detected in the developing embryo during specific developmental periods supporting the current hypothesis that, it may be possible that a limited viral replication occurs in the embryos, but if the embryos have VN antibodies, the VN antibodies prevent the development of viremia in the embryos [19].

Viral antigens were identified only in individual lymphocytes in the cortex of the thymus and infected hemocytoblasts in the bone marrow of tissues collected from commercial broiler breeder chickens by the IPS. However, consistent and observable differences on the intensity of staining were not observed on those positive slides from individual chickens with moderate and high protective antibody titers at the same level of dilution of primary antibodies. Specific staining could not be detected in spleen, liver, duodenum, ovary and oviduct. Most of the tissue sections obtained from commercial broiler breeder chickens and tested positive by the nested PCR assay turned out to be negative in IPS. This could be due to virus replication in those tissues might be limited and below the detection limit of the assay. It might also be related with the age of commercial broiler breeder chickens or the poor sensitivity of the technique compared to the other two detection methods used in the present study namely the nested PCR assay for detecting CAV DNA and ELISA for detection of antibodies against CAV. Smyth *et al*. [17] demonstrated viral antigens in the lymphoid tissues of other organs including proventriculus, the ascending part of duodenum, kidney and lung. However, they also confirmed that, infected cells in these tissues usually cannot be detected for more than 22 days after infection at one day of age.

Comparisons of percentage homologies of the studied CAV isolates with previously characterized local CAV isolates showed diverse similarity among the local isolates. The phylogenetic analysis of 165 deduced amino acid sequences of the VP1 protein also revealed grouping of the Malaysian CAV isolates into two major clusters (Fig. 2). An overall similarity with CAV isolates circulating in south and south-east Asia and Australia was also observed while still having limited variation with isolates from different geographical parts of the world. Natesan et al. [22] also found similar result in that Indian isolate (CAV-E) having maximum similarity with Australian isolate isolate-704, Japanese isolate TR-20 and Malaysian SMSC-1 isolate. Unique amino acid residues observed in isolates from commercial broiler breeder chickens include proline (P) at amino acid position 22 and glutamine (G) at amino acid position 48 in isolates NF4A and PYT4, respectively.

Islam et al. [8] identified amino acid residues at positions 75-I/T, 97-L, 139-Q and 144-Q can be used to group CAV isolates into different groups. In the present study, two distinct groups were observed in the current isolates based on their amino acid profile at these positions. Seven of the isolates from the commercial broiler breeder chickens including previously characterized Malaysian isolate SMSC-1, had 75-I, 97-L, 139-Q and 144-Q and clustered together in cluster I of the deduced amino acid phylogenetic tree. Together in this group also included other isolates from different geographical places. This includes, CIA-1 from USA, CAV-B from India, BD-3 from Bangladesh, isolate 704 from Australia, isolate 130 from Slovenia and NIE/19.04/118 isolate from Nigeria. The remaining five current isolates including previously characterized Malaysian isolates BL-5 [12] and 3-1 [13] had amino acid profile of 75-V, 97-M, 139-K and 144-E. These isolates were found in cluster II of the deduced amino acid phylogenetic tree with other isolates from around the world. In addition, there was no evidence of recombination effect observed in the analysis of Malaysian CAV isolates as reported by Van Santen et al. [23] on CAV isolates from USA. In that study, they indicated that different CAV isolates from Alabama can be divided into two groups with one isolate showing an exceptionally different amino acid profile of I-75, L-97, K-139 and E-144 suggesting of a possible evidence of recombination event.

The analysis of the ratio of synonymous and non-synonymous substitution rate (omega value) indicated the presence of only purifying (negative) selection in the studied isolates. This is similar to the result by Ducatez *et al*. [24] where they indicated a very slow CAV virus evolution at amino acid level corresponding to a strong negative selection (0.04 to 0.20) of VP1 gene in China and worldwide. However, in this study, we found that one of the isolates has omega value of 0.50 meanwhile five out of 12 isolates have omega value between 0.25 to 0.35 and the rest with omega value of 0.07 to 0.11 (Table 2). In our previous study, we suggested that the BL-5 isolate was distantly related to other Malaysian CAV isolates, SMSC-1 and 3-1 [12], has omega value of 1.50 suggesting of a positive selection of VP1 protein in this isolate (Table 2).

The overall phylogenetic pattern and clustering of different CAV isolates based on the partial VP1 gene in this study was similar to previous one based on complete sequence of VP1 gene [24] or the entire CAV genome [25] for the common CAV isolates considered in all the three studies. This suggests that relationships of different CAV isolates can be determined on the basis of partial sequence of VP1 gene due to the fact that most of the amino acid substitutions in comparisons between isolates lies in VP1 gene and more specifically on the N-terminal half of VP1 gene.

## Conclusion

Generally, from the present study we can conclude the widespread occurrence of CAV infection in commercial broiler breeder farms at least in the three states of Malaysia. Detection of significantly higher percentage of positive DNA from spleen, thymus and bone marrow make these organs an excellent choice of organs in the screening and diagnosis of flocks for CAV infection. The finding of CAV DNA in embryos from broiler breeder chickens with neutralizing antibodies supports the previous finding that CAV may remain in the gonads of antibody positive chickens and can be vertically transmitted to their progeny. However, the importance of transmission of viral DNA detected by nested PCR assay still needs further study and explanation. The result also indicated the occurrence of genetic variability in local CAV isolates that can be divided at least into two groups based on characteristic amino acid substitutions at positions 75, 97, 139 and 144 of the VP1 protein. However, the CAV isolates showed only negative selection based on the calculated omega value of the partial sequences of the VP1 gene. The characterized CAV isolates show overall similarity with CAV isolates circulating in South East Asia and Australia while still having limited variations with isolates from different geographical parts of the world.

## Methods

### Broiler breeder farms

Tissue and blood samples were collected from 12 commercial broiler breeder chicken farms located at three states of Peninsular Malaysia. The farms were not vaccinated against CAV and the samples were collected from a total of 60 broiler breeder hens that range in age from 25–35 weeks.

### Sample collection

A total of 420 organ samples were collected. Spleen, thymus, liver, bone marrow, duodenum, ovaries and oviduct were organs collected from each hen. Blood samples were collected from 52 broiler breeder hens by veno-puncture of the wing vein. Sera were separated and stored at -20°C until used.

Ten eggs were also collected from each farm and incubated for 18–20 days. Prior to hatching pooled embryonic organ samples consisting of thymus, bursa of Fabricius and spleen together with egg shell membrane (ESM) were collected from individual embryos. Tissue samples from the hens and embryos were stored at -20°C until DNA extraction.

### DNA Extraction from Samples

DNA was extracted from a total of 420 tissue sample from hens and 52 pooled embryo samples. Briefly, tissue samples (1–5 mg) were homogenized in Phosphate Buffered Saline (PBS) solution by grounding with a mortar and pestle. Then the homogenate (~700 μl) was transferred into 1.5 ml eppendorf tube and centrifuged at 13000 rpm for 1 minute. The supernatant was then transferred into a new microcentrifuge tube. DNA extraction was carried out using MasterPure complete DNA and RNA purification kit (Epicentre, Madison, WI), following the instructions of the manufacturer with some modifications. The concentration and purity of the extracted DNAs were determined by a spectrophotometer (Beckman, USA) according to the method described by Sambrook *et al*. [26].

### Detection of CAV by nested PCR assay

The extracted DNA was first screened for CAV DNA using a highly sensitive nested detection PCR as previously described by Cardona *et al*. [18] with slight modifications. The first-step PCR reaction was carried out using 20 pmol each of the primers O3F and O3R amplifying a 386 bp fragment of the VP3 gene [18]. The PCR reaction was carried out in a total volume of 25 μl using the following cycling parameters: initial denaturation of 94°C for 2 min followed by 35 cycles of denaturation, annealing and extension at 94°C for 2 min, 50°C for 1 min and 72°C for 1 min, respectively, and the final extension was carried out at 72°C for 3 min. An aliquot of the first PCR reaction (1 μl) was then added to 24 μl of a new mastermix (total volume 25 μl) containing 20 pmol of the nested primers N3 and primer N4 for amplification of a 209 bp nested fragment of the VP3 gene as reported by Cardona *et al*. [18]. The nested PCR assay was carried out in MyCycler^® ^Thermal Cycler (Bio-Rad, Hercules, CA, USA). The PCR products were analyzed by 1.8% agarose gel electrophoresis and the photographs were taken using Bio Imaging System in GeneSnap program (SynGene, Cambridge, UK).

### Amplification of partial VP1 gene for sequencing

Spleen samples from each farm that were detected CAV positive by VP3 nested PCR assay were used for amplification of partial VP1 gene using primers VP1F and VP1R for the first round amplification as described by Natesan *et al*. [22]. Nested fragment of first round amplification were amplified using primers O1F and PshA1R [18]. The first round PCR condition was carried out using the following cycling parameters: initial denaturation of 94°C for 4 min followed by 35 cycles of denaturation, annealing and extension at 94°C for 1 min, 57°C for 1 min and 72°C for 2 min, respectively, and the final extension was carried out at 72°C for 8 min. The second synthesis was carried out in a 50 μl reaction mixture with 1 μl of the first PCR reaction product and cycling parameters similar to that described for nested detection PCR. The PCR products were run on 1.6% agarose gel electrophoresis and purified from the gel by using GeneAll^® ^kit (General Biosystem Inc., Korea) following the supplied instructions.

### Sequence and phylogenetic analysis

Using the gel purified PCR products, the partial nucleotide sequences of VP1 gene were determined by direct sequencing in both direction using nested primers O1F and PshA1R. Sequencing reactions were performed in MJ Research PTC-225 Peltier Thermal Cycler using ABI PRISM^® ^BigDyeTM Terminator Cycle Sequencing Kits with AmpliTaq DNA polymerase (FS enzyme) (Applied Biosystems, CA, USA). Each sample was sequenced three times to confirm consistency of the sequencing results.

DNA sequences of the 12 CAV isolates were aligned and compared with 20 local and foreign CAV isolates retrieved from the GenBank database. Sequences of VP1 gene for the studied Malaysian isolates were submitted to GenBank under the following accession numbers: MF1A [FJ167513]; MF3C [FJ167514]; M1B1 [FJ167515]; M3B5 [FJ167516]; NF1D [FJ167517]; NF2C [FJ167518]; NF3A [FJ167519]; NF4A [FJ167520]; P12B [FJ167521]; P24A [FJ167522]; PYT4 [FJ167523]; PPW4 [FJ167524]. The retrieved CAV isolates sequence name, GenBank accession numbers (in square brackets) and country are as follows: Cux-1 [M-M55918], Germany; Cux-1N [NC001427], Germany; SMSC-1 [AF285882], Malaysia; SMSC-1P60 [AF390102], Malaysia; 3-1 [AF390038], Malaysia; 3-1P60 [AY040632], Malaysia; BL-5 [AF527037], Malaysia; BL-5/P90 [AY150576], Malaysia; Isolate 704 [U65414], Australia; CIA-1 [L14767], USA; ConnB [U69548], USA; Del-Ros [AF313470], USA; 26P4 [D10068], USA; China [AF448446]; A2-[AB031296], Japan; BD-3 [AF395114], Bangladesh; CAV-A [AY583755], India; CAV-B [AY583756], India; NIE/19.04/118 [AJ888524], Nigeria; 130 [DQ016138], Slovenia. Percentage of homology, sequence identity matrix and translation from nucleotides to amino acids were determined using BioEdit software package version 7.01 [27]. Multiple sequence alignment of nucleotide and translated amino acids were performed using ClustalX software [28]. The phylogenic analysis of 165 deduced amino acids of the VP1 gene was performed with the software MEGA4 for phylogenetic and molecular evolutionary analyses using the Neighbor Joining Phylogeny reconstruction method with Poisson correction analysis and bootstrap consensus tree inferred from 1000 replicates [29]. Omega values [ratio of non-synonymous (*K*_A_) to Synonymous (*K*_S_) substitution rates] was calculated in comparison to consensus nucleotide sequences using PAL2NAL program [30].

### ELISA

The sera were tested using a commercial ELISA kit (Idexx Lab, USA) at a 1:100 dilution and the results were expressed as S/N ratios (sample to negative ratio) according to manufacturer's instructions. Optical density value was read at 650 nm wave length on an ELX 800™ microplate reader (BIO-TEK Instruments, USA). The ELISA antibody titer has 78% correlation to virus neutralization titers [19].

### Experimental infection of chicks with CAV

Eight 5 days old specific-pathogen-free (SPF) chicks were obtained from Veterinary Research Institute (VRI), Ipoh, Perak, Malaysia. The chicks were divided into 2 groups. Group 1 (n = 5) was inoculated intramuscularly with 1 ml of SMSC-1 CAV isolate cell culture inoculum containing 10^5.5 ^TCID_50_/ml [13]. Group II (n = 3) was left uninoculated as negative control chicks. Each group was reared separately in different room and the chicks were observed daily, and feed and water were provided *ad libitum*. The chicks were sacrificed at 14 days p.i. (post inoculation) for collection of organs. Tissue samples collected from infected and uninoculated chicks were processed and considered as positive and negative control slides for immunoperoxidase staining (IPS), respectively. All experimental research carried out on animals in this paper (including chicken hyperimmune serum production) followed internationally recognized guidelines and approved by animal care and use committee at the Faculty of Veterinary Medicine in University Putra Malaysia (Ref: UPM/FPV/PS/3.2.1.1551/AUP-R4).

### Specimen preparation for immunohistochemical staining

Tissue samples were fixed in 10% (v/v) neutral phosphate-buffered formalin for about 24 hrs and were then trimmed to the thickness of 0.5 cm. The bone marrow samples were decalcified by 5% nitric acid solution following the procedure of Luna [31]. Following tissue processing, tissue blocks were sectioned at a thickness of 4 μm and collected on clean silanized microscope slides [32,33].

### Immunohistochemical staining

Prior to staining, the tissue slides were deparaffinized to remove embedding media and rehydrated following the supplied instructions. Antigen retrieval was achieved using the microwave-based antigen retrieval technique [33,34]. The staining procedure of the detection system was carried out following the manufacturer's instruction manual [DakoCytomation Envision^®^+ Dual Link System-HRP (DAB+), Denmark]. A known negative and positive antigen control and a negative serum control were included in every procedure.

### Chicken hyperimmune serum production

The hyperimmune serum production was carried out in four 60 weeks old SPF broiler breeder roosters obtained from VRI, Ipoh, Perak, Malaysia, with the following immunization protocol: Briefly, at day 0, prior to inoculation, blood samples were collected from all chickens to test their freedom from CAV antibody. Then the roosters were immunized orally with 2 ml of live CAV vaccine AviPro^®^THYMOVAC (Lohmann Animal Health, Cuxhaven, Germany). The immunization regimen was repeated at 14, 28, and 42 days after the first immunization in combination with Freund adjuvant (Sigma, USA). At day 56, blood was withdrawn from all the chickens from the wing vein to separate the hyperimmune serum produced. Commercial Idexx ELISA kit (Idexx Lab, Westbrook, Maine, USA) was used to evaluate the antibody titer of the chicken hyperimmune serum at different levels of immunization.

### IgY purification from chicken hyperimmune serum

IgY purification was carried out using Pierce^® ^Thiophilic Adsorption Kit (Pierce, USA). The T-gel was initially used according to the manufacturer's instruction for mammalian immunoglobulin purification. For the purification of IgY from chicken serum, the manufacturer's instruction was followed together with optimized protocol of T-gel chromatography for the purification of IgY from chicken sera as described by Constantinoiu *et al*. [35].

### Statistical methods

Data from the distribution of CAV DNA in organ samples from commercial broiler breeder chickens were analyzed by Kruskal-Wallis one-way ANOVA with significance defined at *P *< 0.05. Groups with significance difference in means from the rest were determined using Q-statistics [36].

## Competing interests

The isolated Malaysian CAV isolates are currently been modified for development of live attenuated vaccines for poultry.

## Authors' contributions

Design and conception of the study (ARO, ZH, MHB, TCG); samples detection by PCR assays (ZH, ARO, TCG), Immunohistochemstry (ZH, MHB), sequence alignment and phylogenetic tree analysis (ZH, ARO), manuscript preparation (ZH, ARO, MHB); All the authors read and approved the final manuscript.

## Supplementary Material

Additional file 1 **Tissue distribution of CAV DNA in various organs from commercial broiler breeder hens**. Values with the same lowercase superscript are not significantly different (*P *< 0.05) by Kruskal-Wallis one-way ANOVA analysis. The difference in the tissue distribution of CAV DNA between spleen, bone marrow, thymus and ovary was found to be not significant. However, the distribution of CAV DNA in liver, duodenum and oviduct was found significantly less compared to spleen, thymus, bone marrow and ovary. Click here for file
