# Supplementary material for: Detection and characterization of chicken anemia virus from commercial broiler breeder chickens
Source: Virol J. 2008 Oct 27;5:128. doi: 10.1186/1743-422X-5-128 (PMC2605446; doi:10.1186/1743-422X-5-128)
Supplement: Additional file 1 — Tissue distribution of CAV DNA in various organs from commercial broiler breeder hens. Values with the same lowercase superscript are not significantly different (P < 0.05) by Kruskal-Wallis one-way ANOVA analysis. The difference in the tissue distribution of CAV DNA between spleen, bone marrow, thymus and ovary was found to be not significant. However, the distribution of CAV DNA in liver, duodenum and oviduct was found significantly less compared to spleen, thymus, bone marrow and ovary. [file 1743-422X-5-128-S1.pdf]

State	Farm	Age in weeks	No. of hens tested	No. of organs tested	Distribution of CAV DNA in organs (No. CAV positive/total no. of samples)						
					**Spleen**	**Thymus**	**Bone marrow**	**Liver**	**Duodenum**	**Ovary**	**Oviduct**
Melaka	MF1	31	5	35	4/5	3/5	4/5	3/5	2/5	3/5	2/5
	MF3	36	5	35	5/5	4/5	4/5	2/5	3/5	4/5	3/5
	1B	35	5	35	3/5	3/5	4/5	3/5	2/5	4/5	2/5
	3B	35	5	35	4/5	4/5	3/5	3/5	3/5	3/5	3/5
Negeri Sembilan	NF1	25	5	35	4/5	4/5	3/5	3/5	2/5	3/5	2/5
	NF2	25	5	35	3/5	3/5	4/5	2/5	2/5	3/5	3/5
	NF3	30	5	35	4/5	3/5	3/5	3/5	3/5	2/5	3/5
	NF4	34	5	35	3/5	4/5	4/5	2/5	2/5	4/5	2/5
Perak	PPW	30	5	35	4/5	2/5	3/5	3/5	2/5	4/5	2/5
	PYT	35	5	35	3/5	4/5	3/5	3/5	2/5	3/5	2/5
	P12	28	5	35	4/5	3/5	4/5	3/5	3/5	4/5	3/5
	P24	27	5	35	4/5	4/5	3/5	2/5	2/5	3/5	2/5
**Total**			**60**	**420**	**45/60**	**41/60**	**42/60**	**32/60**	**28/60**	**40/60**	**29/60**
**Mean % $\pm$ SD**					**$75.0^{a} \pm 12.4$**	**$68.3^{a} \pm 13.4$**	**$70.0^{a} \pm 10.4$**	**$53.3^{b} \pm 9.8$**	**$46.7^{b} \pm 9.8$**	**$66.7^{a} \pm 13.0$**	**$48.3^{b} \pm 10.3$**
